# Conditions of the Central-Limit Theorem Are Rarely Satisfied in Empirical Psychological Studies

**DOI:** 10.3389/fpsyg.2021.762418

**Published:** 2021-11-08

**Authors:** Tadamasa Sawada

**Affiliations:** School of Psychology, National Research University Higher School of Economics, Moscow, Russia

**Keywords:** the classical central-limit theorem, the Lyapunov/Lindberg central-limit theorem, normal distribution, random sampling, parametric test

## Introduction

Consider randomly sampling variables from an infinite population^1^ and computing their normalized-sum, which is the difference between the average of the variables and the mean of the population multiplied by the square-root of the sample size. The Central-limit Theorem (CLT) assures us that this normalized-sum asymptotically follows a normal distribution when the sample size goes to positive infinity and when the population is with a finite non-zero variance (Dekking et al., 2005; Kwak and Kim, 2017; see also Cuadras, 2002).

Many statistical and analytical methods (e.g., *t*-test, linear-regression, and ANOVA) used in empirical Psychology studies are formulated on the basis of the CLT and some other assumptions (Lumley et al., 2002; Nikulin, 2011; Wijsman, 2011; Kim and Park, 2019). Note that the CLT assumes that the sample size goes to positive infinity but that the sample size is always finite in a real experiment. With the finite sample size, the average approximately follows the normal distribution when the sample size is sufficiently large but the “sufficiently-large” sample size depends on the shape of the distribution of the population (e.g., heavy tails, see Cuadras, 2002; Wilcox, 2012), so, the population should be close to a normal distribution especially when the sample size is small. Even if the distribution of the average of the finite samples is approximately-normal, a discrepancy of this distribution from the normal distribution can substantially affect the results of one's statistical and analytical methods (Wilcox, 2012). Note also that some other statistical and analytical methods assume that the population itself is normally distributed, e.g., some Bayesian models.

In many Psychology studies, the population represents a group of people^2^ and the samples represent individual participants sampled from the group and they are often the averages of the responses of the individual participants. Namely, the sampling procedure used in these studies is conducted in 2 steps: (i) it samples the participants in the group and (ii) it samples the responses of each participant. It is often believed that the population, itself, can be regarded as approximately following a normal distribution based on the CLT (Bower, 2003; Miles and Banyard, 2007; Sotos et al., 2007). The population in these studies are actually a distribution of the “averages,” which is the sum of the normalized-sum divided by the square-root of the sample size and the population mean, but these averages were computed from the responses of individual participants and this fact violates conditions in the CLT.

In this study, I describe how the conditions in the CLT are usually *not* satisfied in empirical Psychological studies by comparing the formulation of the CLT with a common experimental procedure used in empirical Psychological studies. This explains why the CLT *cannot* assure that the population follows a normal distribution no matter how large the sample size is in these studies. This applies regardless of the number of participants or the number of trials run by each participant.

## The CLT and a Procedure Commonly Used in Empirical Psychological Studies

**Figure 1:**
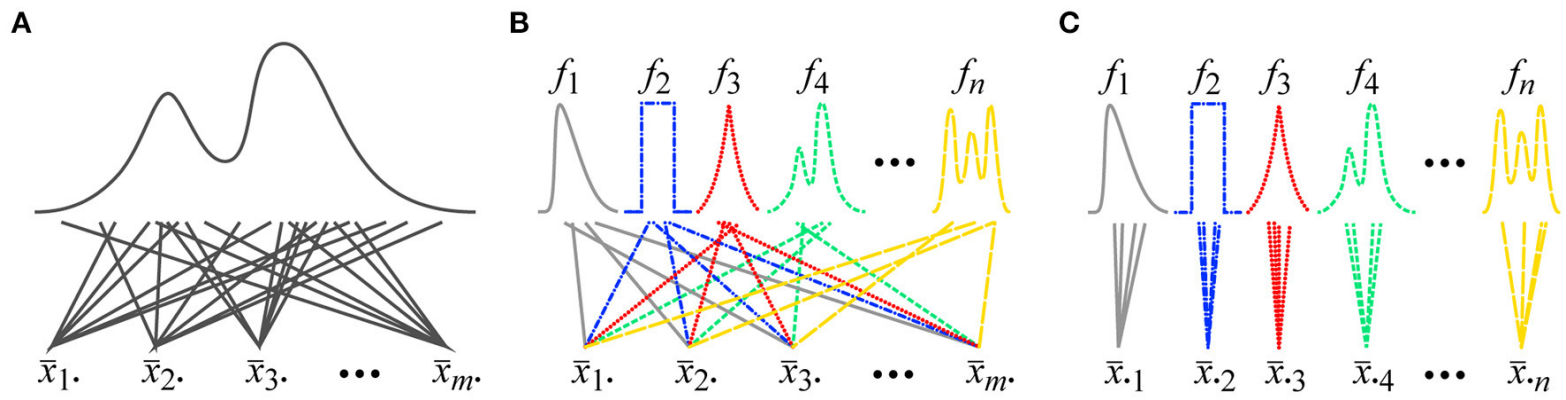
**(A)** A procedure for sampling data for the classical CLT. In a session for the classical CLT, a fixed number of variables are sampled from a single arbitrary distribution and the normalized-sum of the *n* sampled variables is computed. **(B)** A procedure for sampling data for the Lyapunov/Lindberg CLT. In a session for the Lyapunov/Lindberg CLT, a single variable is sampled from each of *n* arbitrary distributions and the normalized-sum of the *n* sampled variables is computed. **(C)** A procedure commonly used for sampling data in empirical Psychological studies. Individual participants can be represented by distributions *f*_1_, *f*_2_, …, *f*_*n*_. A fixed number of variables are sampled and the normalized-sum of the sampled variables is computed for each distribution (Note that the distributions in this figure are clearly non-normal. This violation of the assumption of normality has been exaggerated in this figure to enhance the clarity of its explanation).

In Psychology, one specific type of the CLT is described in almost all of the Statistics textbooks and this type is referred to as the *classical* CLT. Consider that an arbitrary distribution with a finite non-zero variance is given, and that random variables (*x*_11_, *x*_12_, …, *x*_1*n*_) are sampled from this distribution for *n* times, and their normalized-sum ${\overset{\;}{\bar{x}}}_{1\cdot }$ (the difference between the average and the population mean multiplied by $\sqrt{n}$) is computed (Figure 1A). This session is repeated for *m* times. This sampling is independent and the sampled variables do not depend on one another. Once this is done, the normalized-sums (${\overset{\;}{\bar{x}}}_{1\cdot }$, ${\overset{\;}{\bar{x}}}_{2\cdot }$, …, ${\overset{\;}{\bar{x}}}_{m\cdot }$) from the *m* sessions can be asymptotically regarded as variables sampled from a normal distribution when *n* goes to infinity.^3^

Now, consider a procedure commonly used in empirical Psychological studies. Some dependent variable is measured in multiple trials and an average of the measured variable is computed for each participant (Figure 1C). These averages were collected from multiple participants. Note that the distribution of the dependent variable is different across the participants because of their individual differences (Williams, 1912). These distributions are also different from the distribution of the population of the participants. These facts violate the conditions of the identically-distributed random variables for the *classical* CLT.

A case in which variables are sampled from multiple distributions is discussed in the *Lyapunov/Lindberg* CLT (Billingsley, 1995; Petters and Dong, 2016). Consider that *n* arbitrary distributions (*f*_1_, *f*_2_, …, *f*_*n*_) with finite non-zero variances are given and that a single random variable ${\bar{x}}_{1i}$ is independently sampled from each distribution *f*_*i*_ (*i* = 1, 2, …, *n*), and the normalized-sum ${\overset{\;}{\bar{x}}}_{1\cdot }$ of the *n* sampled variables (*x*_11_, *x*_12_, …, *x*_1*n*_) is computed (Figure 1B). This session is repeated *m* times. These distributions can be different from one another. This sampling is independent and the sampled variables do not depend on one another. Once this is done, the normalized-sums (${\overset{\;}{\bar{x}}}_{1\cdot }$, ${\overset{\;}{\bar{x}}}_{2\cdot }$, …, ${\overset{\;}{\bar{x}}}_{m\cdot }$) from the *m* sessions can be asymptotically regarded as variables sampled from a normal distribution when *n* goes to infinity and when the distributions *f*_1_, *f*_2_, …, and *f*_*n*_ satisfy Lindberg's condition about their variances:


$$\underset{n\to \infty }{\lim}\frac{1}{\sigma_{\Sigma }{\;}^{2}}\sum_{i=1}^{n}𝔼\left({\left(x_{i}-\mu_{i}\right)}^{2}\left[\left|x_{i}-\mu_{i}\right|>\varepsilon \sigma_{\Sigma }\right]\right)=0$$

$$\begin{array}{l} {\sigma_{{\;}_{\Sigma }}}^{2}=\overset{n}{\underset{i=1}{\sum }}𝔼\left({\left(x_{i}-\mu_{i}\right)}^{2}\right) \end{array}$$

$$\left[\left|x_{i}-\mu_{i}\right|>\varepsilon \sigma_{\Sigma }\right]\text{:=}\{\begin{array}{cc} 1 & \;if\;\left|x_{i}-\mu_{i}\right|>\varepsilon \sigma_{\Sigma }\\ 0 & \;\text{else} \end{array}$$

where ε is a free parameter, *x*_*i*_ is a random variable from the distribution *f*_*i*_, 𝔼(*x*) is an operator computing an expected value of a random variable *x*, μ_*i*_ = 𝔼(*x*_*i*_) is the mean of *f*_*i*_, and $𝔼\left({\left(x_{i}-\mu_{i}\right)}^{2}\right)$ is the variance ${\sigma_{i}}^{2}$ of *f*_*i*_. The parameter ε is an arbitrary non-zero positive number and it is fixed during the limit *n* → ∞. Lindberg's condition is a sufficient condition for the *Lyapunov/Lindberg* CLT. Lindberg's condition implies that the individual variances ${\sigma_{1}}^{2}$, ${\sigma_{2}}^{2}$, …, and ${\sigma_{n}}^{2}$ of the distributions *f*_1_, *f*_2_, …, and *f*_*n*_ become negligibly small when they are compared with the sum of these variances ${\sigma_{{\;}_{\Sigma }}}^{2}$ as *n* → ∞ (Petters and Dong, 2016). Note that Lindberg's condition cannot be strictly satisfied in a real experiment because *n* is finite, but the condition can be brought closer to being satisfied when none of the variances ${\sigma_{1}}^{2}$, ${\sigma_{2}}^{2}$, …, and ${\sigma_{n}}^{2}$ is very much larger than the other variances.

The *Lyapunov*/*Lindberg* CLT also does not validate the assumption of normality using a procedure commonly used in empirical Psychological studies. Recall that some dependent variable is measured in multiple trials and an average of the measured variable is computed for each participant in the common procedure (Figure 1C). There are the participants' individual differences and the distribution of the dependent variable is different across the participants. Namely, the averages are computed *within* the individual distributions of the participants in the common Psychological procedure. But note that, according to the *Lyapunov*/*Lindberg* CLT, the averages should be computed *across* the distributions (Figure 1B). The common Psychological procedure does not follow the procedure of the *Lyapunov/Lindberg* CLT.

Note that the common procedure used in empirical Psychological studies can be modified so that the conditions of the *classical* or *Lyapunov/Lindberg* CLT are better satisfied (see Hoefding, 1951; Hájek, 1961). Some dependent variable is measured in multiple trials and an average of this measured variable is computed for each participant in the common procedure. Let *x*_*ji*_ be the measured variable in the *j*-th trial of the *i*-th participant. The average of the measured variables of this participant is computed as ${\overset{\;}{\bar{x}}}_{\cdot i}=t^{-1}\underset{j}{\sum }x_{ji}$ where *t* is the number of trials. Once this is done, the averages from the *n* participants were randomly categorized into *s* sets that have an equal number (*n*/*s*) of the averages, and the averages within each group are also averaged:


$$\begin{array}{l} {\overset{\;}{\bar{\bar{x}}}}_{k}=\frac{s}{n}\underset{{\overset{\;}{x}}_{\cdot i}\in S_{k}}{\sum }{\overset{\;}{x}}_{\cdot i} \end{array}$$

where *s* is one of divisors of *n* and *S*_*k*_ (*k* = 1, 2, …, *s*) is the *j*-th set of the averages. If the number *n*/*s* of the participants in each set is sufficiently large, this modified procedure better satisfies the conditions of the *classical* CLT than the common procedure used in empirical Psychological studies (see https://osf.io/kn8mh/ for a computer simulation of this modified procedure).

For the *Lyapunov/Lindberg* CLT, an average of the measured variables (*x*_*ji*_ in the *j*-th trial of the *i*-th participant) is computed *across* the participants for each order number of the trials:


$$\begin{array}{l} {\overset{\;}{\bar{x}}}_{j\cdot }=\frac{1}{n}\overset{n}{\underset{i=1}{\sum }}x_{ji} \end{array}$$

This modified procedure better satisfies the conditions of the *Lyapunov/Lindberg* CLT than the common procedure used in empirical Psychological studies when the number of the participants *n* is sufficiently large, and when Lindberg's condition is satisfied (see https://osf.io/kn8mh/ for a computer simulation of this modified procedure).

## Discussion

This study explained how conditions in the central-limit theorem (CLT) are usually *not* satisfied in empirical Psychological studies. The population usually represents a group of people in these studies and when it does, the CLT cannot assure one that that the population follows a normal distribution no matter how large the sample size is. The study also discussed two possible modifications of a procedure commonly employed in the studies to better satisfy the conditions of the CLT (see https://osf.io/kn8mh/ for computer simulations of these modified procedures).

Some commonly used parametric statistical tests, such as the *t*-test and the ANOVA (see also Tan and Tabatabai, 1985; Fan and Hancock, 2012; Cavus et al., 2017 for the robust-ANOVA) are robust to some extent against some types of non-normality of the population (see Lumley et al., 2002 for a review) but not against some other types of non-normality, e.g., heavy tails and outliers (Cressie and Whitford, 1986; Wilcox, 2012). Note that there are some non-parametric statistical tests that do not use a normality assumption for the population but these non-parametric tests are not universally more robust than the parametric tests. These non-parametric tests use some other assumptions about the data, such as equal variance, just as the parametric tests do. These non-parametric tests can be affected more subtlety than the parametric tests when these assumptions are violated (e.g., Fagerland, 2012, see also Algina et al., 1994).

The assumption of normality is also used in Bayesian statistics. The Bayesian alternatives used in conventional statistical tests often use Bayes factors (BFs) instead of the *p*-values used in the conventional tests. These BFs correlate well with the *p*-values (Rouder et al., 2012; Johnson, 2013; see also Francis, 2017), so the BFs can be robust to some extent just as the *p*-values are when the normality assumption is violated. There are studies that use Bayesian statistical models to explain their results. These models are composed of multiple parts with independent probability distributions. These distributions are often assumed to be normal. The validity of this assumption is difficult to test especially when their parts represent some variables that are not directly observable. The robustness of these models against the violation of the normality assumption should depend on the structures of the models but their structures are different from one another, so the effect of their model structure and the robustness of the model need to be studied systematically.

The assumption of normality is fundamental in many statistical analyses that are used in empirical Psychological studies but this assumption is rarely assured by the CLT. The conventional statistical analyses should be regarded as being, at best, descriptive. Experimental Psychologists should check their row data and should discuss “effects” only if the effects are clear in their data. If they want to make inferences based on the results of statistical analyses, more modern statistical methods should be considered: e.g., Robust statistics (Tan and Tabatabai, 1985; Huber and Ronchetti, 2009; Fan and Hancock, 2012; Wilcox, 2012; Cavus et al., 2017).

## Author Contributions

The author confirms being the sole contributor of this work and has approved it for publication.

## Conflict of Interest

The author declares that the research was conducted in the absence of any commercial or financial relationships that could be construed as a potential conflict of interest.

## Publisher's Note

All claims expressed in this article are solely those of the authors and do not necessarily represent those of their affiliated organizations, or those of the publisher, the editors and the reviewers. Any product that may be evaluated in this article, or claim that may be made by its manufacturer, is not guaranteed or endorsed by the publisher.

## Footnotes

^1^This study, for the sake of simplicity, only the case in which the population was infinite was considered.

^2^This group is assumed to be sufficiently large for its population to be regarded as being infinite. Otherwise, the population is finite and the participants from the group can be regarded as samples from the finite population without replacement. The CLT cannot be applied under this condition (Plane and Gordon, 1982). This happens when a study is about a small group of people, e.g., patients with a rare disease.

^3^The asymptotic normality of the *classical* CLT, of the *Lyapunov/Lindberg* CLT, and of the two modified procedures proposed in this study are simulated computationally (https://osf.io/kn8mh/).
